# Effect of right hemispheric damage on structured spoken conversation

**DOI:** 10.1371/journal.pone.0271727

**Published:** 2022-08-11

**Authors:** Yeo Jin Kim, Hye Yeong Jeong, Hui-Chul Choi, Jong-Hee Sohn, Chulho Kim, Sang-Hwa Lee, Joon Soo Shin, So Ra Chin, Yoon Kyoung Lee, So Jung Oh, Ji Hye Yoon

**Affiliations:** 1 Department of Neurology, Chuncheon Sacred Heart Hospital, Chuncheon, Korea; 2 Department of Speech Pathology, Graduate School of Health Sciences, Hallym University, Chuncheon, Korea; 3 Department of Speech-Language Pathology and Audiology, Graduate School of Hallym University, Chuncheon, Korea; 4 Division of Speech Pathology and Audiology, Research Institute of Audiology and Speech Pathology, College of Natural Sciences, Hallym University, Chuncheon, Korea; 5 Department of Communication Disorders & Audiology, Tongmyong University, Busan, Korea; The University of Edinburgh, UNITED KINGDOM

## Abstract

Patients with right hemisphere damage (RHD) occasionally complain of difficulties in conversation. A conversation is a type of communication between the speaker and listener, and several elements are required for a conversation to take place. However, it is unclear which of those elements affect communication in patients with RHD. Therefore, we prospectively enrolled 11 patients with right hemispheric damage due to acute cerebral infarction, within 1 week of onset. To evaluate patients’ conversational abilities, we used a structured conversation task, namely, the “Hallym Conversation and Pragmatics Protocol”. The topics of conversation were “family”, “leisure”, and “other/friends”. The conversation characteristics were classified according to three indices: the “conversational participation index”, “topic manipulation index”, and “conversational breakdown index”. Patients with RHD were compared with 11 age-, sex-, and years of education-matched healthy adults. The most common site of damage in the patients with RHD was the periventricular white matter. There was no significant difference in performance between the two groups according to the conversation participation index and in the discontinuance rate assessed with the conversational breakdown index. However, patients with RHD showed a lower topic maintenance rate and higher topic initiation and topic switching rates, according to the topic manipulation index. Therefore, we explored the characteristics of impaired conversation abilities in patients with RHD by assessing their ability to converse and manage topics during structured conversations, and found difficulties with pragmatics and communication discourse in these patients.

## Introduction

The human brain consists of the left and right hemisphere, and each is involved in the functioning of the brain with different dominance. Generally, in right-handed individuals, the left hemisphere is known as the dominant hemisphere; herein, the language function is predominant. However, patients with right hemisphere damage (RHD) occasionally complain of communication difficulties, despite preservation of normal basic language abilities [1]. The challenges faced by these patients have therefore garnered lesser attention than those of patients with lesions in the left hemisphere. Although several earlier studies have reported communication and conversation disorders in patients with RHD, the characteristics of communication and conversation in patients with stroke in the right hemisphere have been inconclusive [2–4]. We therefore investigated such conversational disorders in patients with acute cerebral infarction with RHD.

### Conversational disorders

A conversation is one of the ways people naturally communicate, and it can occur between two or more speakers and listeners [5, 6]. During social communication, an individual may socially engage with others in a variety of ways, such as by making eye contact, paying attention to common themes, and sharing information and feelings. In order to conduct a conversation, assuming one’s clear intent to participate in the conversation, the following two elements are required. First, in “conversational turn-taking” [7, 8] with the other party, a conversation should alternate between the speaker and the listener [9]. The speaker and listener should not be frequently disturbed, such as by being interrupted while it is their turn to speak [10, 11]. Second, engaging in a conversation regardless of whether it is structured or natural would require topic manipulations skills [12]. This capability includes sub-elements such as initiating, maintaining, and altering conversational topics [8, 13, 14].

Previous studies have examined the effects of RHD due to stroke or trauma on normal discourse, including conversations [3, 4, 15]; one study reported that patients with RHD might be unable to effectively take into consideration and appreciate the listener and fail to maintain the topic appropriately [8]. They also start or end conversations abruptly, perhaps due to an inability to take turns [16–18]. Other studies have shown that patients with lesions in the right hemisphere showed difficulty in understanding the intended meaning of an utterance by grasping only the literal meaning, without understanding the implications at a figurative level (metaphors, idioms, proverbs) of conversations [19].

However, these previous studies rarely involved structured conversations. Their results were obtained by conducting relatively free conversations with participants. Conversation tasks can be useful indicators of communication because they allow us to look at the use of language in context, better than that in procedural or narrative discourses, thereby enabling us to understand the characteristics of speech produced by the individual [20]. However, in free conversation-tasks, the quantitative and qualitative aspects of conversation may be affected by the reactions or topics introduced by the conversation partner. For this reason, using free conversation tests to evaluate language processing in patients with RHD comes with limitations in reliability and validity [3]. Therefore, we conducted a structured conversation task as a basis for evaluating patients with right hemisphere lesions, whose language function was not impaired according to a general language assessment.

### Patients with acute cerebral infarction with RHD

Assessing brain function through stroke lesions is one of the traditionally used methods in the investigation of brain function. When a stroke occurs, the part of the brain affected by the stroke loses its function, which allows us to assume the function of the part where the stroke occurred, based on the patient’s symptoms [21]. However, there are several points to consider when investigating brain function through stroke. First, cognitive deficit profiles differ according to the structure damaged by the stroke. Since many cognitive functions are located in the cortex, cognitive impairment is more likely to occur in stroke with cortical involvement [22]. In addition, a previous study found that cortical lesions after right hemisphere stroke were associated with lower scores in the picture-description interpretive unit measure than subcortical lesions [2]. However, cognitive impairment also occurs when there is only subcortical involvement, but the pattern is different from that with cortical involvement. More complex cognitive deficits, such as executive function deficits in subcortical lesions, appear because a broader network is affected [23]. Second, the cognitive deficits caused by stroke, and its associated symptoms, can change over time because of the plasticity of the brain [24]. During the initial stages of stroke recovery, that is, in the first 1–4 weeks, restoration of brain function begins to appear through structural and functional changes in the area affected by the stroke [25]. Indeed, in a patient with a right hemispheric stroke, a significant difference in a few discourse characteristics were observed 1 month after the stroke, but the difference disappeared 6 months after the stroke [2]. This means that results may change depending on when a test is performed after a stroke, and a deficit that is initially seen might not be so obvious 1 week after a stroke [25]. Finally, the type of stroke also affects brain function. In a previous study, hemorrhagic stroke led to poorer neurologic outcomes than ischemic stroke [26]. For this reason, we only included patients with ischemic stroke (cerebral infarction) in this study.

We conducted a structured conversation task-based study on patients with acute cerebral infarction in the right hemisphere within 1 week of onset, in order to provide a basis for evaluating patients with right hemisphere lesions, whose language function is not impaired according to a general language assessment. The aim of this study was to compare the characteristics of conversation in patients with acute cerebral infarction with lesions in the right hemisphere with those of a healthy control group. In addition, we also describe the lesions that our patients presented with, in order to examine the relevance of these lesions in the impairment of conversational characteristics.

## Material and methods

### Study design and participants

This study included 11 patients (4 men and 7 women) whose right hemisphere was damaged due to cerebral infarction (RHD), who visited the Department of Neurology at a hospital located in Chuncheon, Republic of Korea. In addition, we analyzed patient subgroups by dividing the RHD group into those with damage only to the subcortical area (subcortical group, n = 6) and those with damage to both the cortical and subcortical areas (cortical group, n = 5). Eleven additional age-, sex-, and years of education-matched healthy adults (HA) were enrolled in the study as controls. The inclusion criteria for all participants were: 1) Korean language was their mother tongue; 2) no motor speech disorders and normal hearing and vision; 3) normal Korean-Mini Mental State Examination (K-MMSE) [27] results according to the age and years of education; 4) normal scores on the short form of the Geriatric Depression Scale (SGDS) [28] (8 points or less); 5) right-handed as per the Edinburgh Handedness Inventory (EHI) [29]; 6) normal oral language index according to age and years of education on the Screening Test for Aphasia and Neurologic-communication Disorders (STAND) [30]; and 7) no mental or neurological problems that could affect cognitive performance according to the Health Screening Questionnaire (HSQ) [31]. The additional criteria for the RHD patient group were: 1) diagnosed with acute-stage (within 7 days of post-onset time, POT) right hemisphere cerebral infarction by a neurologist [32]; and 2) stable vital signs and stable neurological states while being monitored by the neurologist. No participant had any medical condition that could affect brain function, and no one was on any medication. All patients had left-sided motor weakness at the time of admission. In most cases, active movement against gravity with some resistance was possible. Only two patients had severe motor weakness, and were only capable of muscle contraction at the time of admission. However, these patients recovered to some degree the next day, and the extent of their weakness was such that an active movement against gravity with some resistance was possible by the time of the structured conversation task. Two patients suffered from the neglect syndrome and one patient had a left-sided visual-field defect at the time of the structured conversation task. Eight out of 11 RHD patients received physical therapy, and four of them received occupational therapy as well, but none of them received speech-language therapy. This research was approved by the Institutional Review Board of Chuncheon Sacred Heart Hospital (IRB#: 2018-03-029). Each participant provided written informed consent before participation, and the study was conducted in line with the World Medical Association Declaration of Helsinki published on the website of the Journal of the American Medical Association.

### Screening tests

In order to assess the basic cognitive abilities of the participants, we conducted the K-MMSE, which is a screening test consisting of 30 points and takes about 10 minutes to complete. The K-MMSE consists of items that determine an individual’s orientation to time and place, attention, memory, language ability, calculation ability, and visuospatial ability, with a higher score indicating a higher cognitive ability. Since depression may affect an individual’s performance on cognitive and language tests and may also be closely related to the person’s degree of participation in conversational situations, we confirmed the degree of depression of our participants with the SGDS, which consists of 15 points, with higher scores indicating greater severity of depression. In order to assess the basic language abilities of our participants, the STAND, which is made up of 20 points and takes about 10 minutes to complete, was implemented. The STAND includes subdomains of spontaneous speech, auditory comprehension, repetition, and naming. The higher the score, the higher the language ability. The EHI was conducted to identify the dominant hemisphere of the patients with RHD, and the HSQ was conducted to confirm that there were no other mental or physical problems affecting the cognitive and language abilities of our participants.

### Conversation task materials and procedure

According to research on elicitation methods for conversational data from clinical groups [8], it seemed inadvisable to rely solely on data from “natural” conversations between a speaker and a partner, because results might depend on the topic of conversation [33]. If topics vary widely, it is difficult to establish how far the content of the conversation (as opposed to the characteristics of the individual) has contributed to the variability. With respect to the materials for eliciting conversations, for instance, certain patients may mask difficulties by keeping the conversation focused on familiar topics, rather than having to listen to and interact with the conversational partner. To prevent this, a conversation was developed around a set of photographs was used. Moreover, the result of such a conversational assessment might depend on the responses of the partner. Some partners may try harder to elicit more utterances or provide more content or topics, while others may not and merely follow along. Previous studies on patients with brain injury [34, 35] showed that having a more consistent conversational partner is desirable in some way.

Based on such previous studies, we used the “Hallym Conversation and Pragmatics Protocol” [7, 36]. This protocol provides a structured procedure to acquire reactions from participants and minimizes the influence of the examiner’s response on the conversation. In order to rule out as much contextual engagement as possible, we instructed the examiner to have minimal engagement. As a consequence of this approach, a patient’s utterances may feel like a monologue/personal narrative. However, the examiner continuously and consistently intervenes from the beginning to the end, which creates a pragmatic conversational situation between the examiner and the subject. Therefore, we describe this task as a “structured conversation”. This protocol has been used for children with autism or language learning disorders, as well as for typically developing children and adolescents [36–39]. Recently, it has also been used as a research tool to confirm the conversational abilities of patients with frontotemporal dementia and has been assessed for its applicability to adults [40]. In order to accurately implement this protocol, the examiner (H.Y.J.) received at least five training sessions (1 hour per session) from the supervisor (Y.K.L) on being a conversation partner. The examiner had majored in speech-language pathology in graduate school and had more than 2 years of clinical experience at the time of the study. Prior to the study, the examiner also practiced collecting conversation data from 10 participants receiving feedback from the supervisor.

The topics of conversation were, in sequence, “family”, “leisure”, and “other/friends”, which are all relevant to daily life. In order to facilitate the initiation of a conversation, four related photos were prepared for the “family” and “leisure” topics, and the photos were placed in separate envelopes according to the topic. The photos used in this task were not provided with the intent to force participants to look and describe exactly what they saw. Rather, based on the procedure of a previous study for conversation sampling [33], we used the photos as a medium to ignite the participant’s interest in the topic and help them start a conversation about it. Participants were fully informed that they were not to describe the pictures before the start of the examination. Each set of pictures was only provided for 30 seconds and then set aside so that the conversation could proceed. Since the participants were sufficiently familiar with the protocol after the first two topics (family and leisure), conversations about “other/friends” were initiated without photographs. All photographs provided were 170 mm long and 220 mm wide. All speech collected through conversation was recorded using an MP3 recorder (ICD-TX 800, SONY).

At the start of the session, the examiner took 10 minutes to build rapport with the participant by asking them simple questions (e.g., “How are you feeling today?”). Thereafter, the examiner as a conversational partner explained the task instructions, and, if the participant was confirmed to have understood the task, the examiner provided two envelopes containing the pictures for the topics related to family and leisure, and the participant randomly selected one envelope (topic) first. The examiner then confirmed that the participant had seen all four photographs for each topic, set aside the photographs, and provided an opportunity for the participant to initiate a conversation. If the participant did not initiate the conversation within 3 seconds, the examiner made a comment regarding the topic, saying “So we will talk about family life/leisure activities”. If there was no response despite mentioning the topic, a related question was asked (e.g., “How is your family life?”). If the participant continued the conversation, the examiner responded with neutral responses (“Okay”, “Yes”) [41, 42]. If the conversation did not continue or if there was a pause longer than 3 seconds, the examiner repeated the participant’s last sentence with a raised intonation without adding any words or sentences, to suggest to the participant that the conversation was still ongoing. This procedure is one of the techniques often used in conversational situations or pragmatic typology to induce speech without providing additional information [43]. Subsequently, if the conversation did not continue or if there was a pause longer than 3 seconds, speech was encouraged with cues such as “And?” and “Oh?”. When the participant showed an utterance or action indicating the end of the topic (e.g., “I have nothing more to say on this topic”), the examiner moved to the next topic to start a similar conversation. After seeing the pictures and talking about the two topics, the examiner asked three questions (“What do you usually do when you meet friends or colleagues these days?”, “What is the happiest thing for you these days?”, “What TV programs do you enjoy these days?”) related to the topic other/friends. The procedure for the last topic was identical to the one described for the first two topics, and the conversation task was completed when all three topics had been discussed.

### Conversation data transcription and analysis

All the collected conversation data were transcribed within 1 week. Based on the criteria used in a previous study [9], unintelligible utterances, meaningless sounds, or automated speech (e.g., counting) were excluded. To achieve consensus, the data transcribed by the inspector (H.Y.J.) were additionally reviewed and confirmed by the corresponding author (J.H.Y) and the author who developed the protocol (Y.K.L). The collected utterances were classified according to the categories described in previous studies [36, 37, 44, 45]. Based on previous studies on pragmatics [39, 40], our analysis was divided into the “conversational participation index”, “topic manipulation index”, and “conversational breakdown index”.

The conversational participation index reflects the extent of engagement in the conversation and is based on the assumption that conversational turns include more than one utterance. Two sub-indices are used: the number of turns, and number of utterances per turn. The number of utterances per turn describes the amount of speech the participant generates in one conversation turn, and reflects how much he or she talks in general. For the latter, the total number of utterances is divided by the total number of turns.

The topic manipulation index measures how often a new topic is initiated or changed during a conversation when the concept, vocabulary, and components are equally connected to the previous topic. This index includes all topics covered by researchers and participants during the completion of the dialog protocol. Five sub-indices are used: number of topics, number of turns per topic, percentage of topic initiation, percentage of topic maintenance, and percentage of topic switching. The number of turns per topic refers to the number of utterances produced per topic, and is calculated by dividing this number by the total number of topics that are discussed until the dialog protocol is completed. Topic initiation occurs when the participant speaks first when inducing a dialogue. The topic initiation rate is measured by dividing the total number of topics initiated by the total number of turns. Topic maintenance means that the participant continues the conversation by adding related content or information in response to the questions or contents of the other party. The overall topic maintenance rate is measured by dividing the total number of topics maintained by the participant by the total number of turns. Topic switching refers to the topic of conversation changing to a new subtopic that has not appeared in previous utterances while the previous turn is still ongoing. The topic switching rate is measured by dividing the total number of topic changes by the total number of turns.

The conversational breakdown index assesses conversation interference. Two sub-indices are used: percentage of overlap and percentage of discontinuance. Conversation overlap occurs when one partner interferes with the other person’s words during the conversation (the participants intervene when the examiner speaks). The conversation overlap rate is measured by dividing the total number of times the topic overlaps during the conversation by the total number of turns. Conversation discontinuance is a case of failure to respond immediately to the other person’s words, delay of more than 3 seconds, abnormal long pause, or no response at all. The rate of discontinuance of a conversation is measured by dividing the total number of discontinuances that occurred during the conversation by the total number of turns. Each measurement of conversation performance is presented in Table 1. The definitions of utterance, turn, and topic are provided in S1 Table in S1 File.

**Table 1:** Measurements of conversation performance.

Index	Sub-index (definition)	Measurement
**Conversational participation indexes**	Number of turns	Total number of turns
	Number of utterances per turn (refers to the amount of speech the subject generates in one conversation turn)	Total utterances/Total number of turn
**Topic manipulation indexes**	Number of topics	Number of total topics
	Number of turns per topic (refers to the amount of utterances produced per topic)	Total topics/Total number of turn
	% of topic initiation (refers to the subject speaks first when inducing a dialogue)	(Total topic initiation/Total number of turn)×100
	% of topic maintenance (refers to continuing the conversation by adding related content or information in response to the questions or contents of the other party)	(Total topic maintenance/Total number of turn)×100
	% of topic switching (refers to the topic of conversation changing to a new subtopic that has not appeared in previous utterances while continuing the previous turn)	(Total topic switch/Total number of turn)×100
**Conversational breakdown indexes**	% of overlap (refers to when one partner interferes with the other person’s words during the conversation)	(Total overlap/Total number of turn)×100
	% of discontinuance (refers to a case of failure to respond immediately to the other person’s words, delay of more than 3 seconds, abnormal long pause, or no response at all)	(Total discontinuance/Total number of turn)×100

### Reliability

To confirm the inter-rater reliability of measurements, we calculated the agreement between the data analysis of two analysts. The first analyst was the examiner (H.Y.J) with a major in speech-language pathology and more than 2 years of clinical experience at the time of the study. The second analyst was a Master’s student majoring in speech-language pathology, who was also a certified speech-language pathologist. The first and second analysts received training in conversational procedures and analytical methods. A random sample comprising 20% of the data was selected and analyzed, and the analytical reliability was found to exceed 90%. The agreement rate was assessed for the three indices, namely the conversational participation index, the topic manipulation index, and the conversational breakdown index. The rates of agreement were as follows: 1) conversational participation index, 97.17% (number of turns: 97.21% and number of utterances per turn: 97.14%); 2) topic manipulation index, 96.83% (number of topics: 97.15%, number of turns per topic: 97.45%, topic initiation: 97.52%, topic maintenance: 96.51%, and topic switching: 95.56%); and 3) conversational breakdown index, 98.08% (overlap: 98.26% and discontinuance: 97.91%).

### Magnetic resonance imaging

Standardized T1, T2, fluid attenuated inversion recovery, and diffusion-weighted images (DWIs) were acquired for all participants at the Chuncheon Sacred Heart hospital using the same 3.0 T magnetic resonance imaging (MRI) scanner (Philips 3.0T Ingenia, Koninklijke Philips Electronics N.V., Amsterdam, Netherland). Images were obtained in one session for all participants, and all MR images were obtained in the same orientation and slice positions. DWIs were obtained using the following parameters: axial slice thickness of 3.0 mm, inter-slice thickness of 4.5 mm, repetition time of 3980 ms, echo time of 84 ms, flip angle of 90°, and matrix size of 256 × 256 pixels.

### Visualization of infarcted lesions on a transparent brain

To illustrate the distribution of infarcted lesions, axial slices from DWIs of each patient were manually marked on the high-definition structural brain template using MRIcro software (http://www.mccauslandcenter.sc.edu/mricro/; Chris Rorden, Columbia, SC, USA) Axial slices were matched to the Montreal Neurological Institute T1 template within the MRIcro software program. The lesions, drawn as regions of interest for each patient, were displayed on a common template in order to determine areas of lesion overlap.

### Statistical analyses

To compare performance according to the conversational participation index (number of turns and number of utterances per turn), topic manipulation index (total number of total topics, number of turns per topic, rate of topic initiation, rate of topic maintenance, and rate of topic switching), and conversational breakdown index (rate of overlap and rate of turn discontinuance) between groups, the Kolmogorov-Smirnov and Shapiro-Wilk tests were used first to test for normality. The results of this analysis showed that a few measurements (rate of overlap and rate of turn discontinuance in the HA group) did not satisfy the normal distribution assumption. In addition, because the number of samples included in each group was small, the Mann-Whitney U test, a nonparametric test, was performed for all measurements. Statistical Package for the Social Sciences version 22 (IBM Corp., Armonk, NY, USA) was used for all statistical analyses.

## Results

### Baseline characteristics and lesion distribution

There was no significant difference in age, sex, or years of education between the HA and RHD groups. The SGDS, K-MMSE, and STAND scores were also not significantly different between the HA and RHD groups. In addition, in terms of the impaction of the visuospatial neglect, we observed that no RHD patients had neglect symptoms through bedside observation. We also confirmed that 10 out of 11 RHD patients had no problems with the interlocking pentagon drawing item included within the MMSE. Participant information is provided in Table 2 and more detailed information on patients with RHD is presented in S1 Appendix. The mean POT of the patients with RHD was 3.82 (± 2.32) days.

**Table 2:** Demographic information and test scores for the patient and control groups.

	RHD	HA	*P*
**Age (yr)**	67.27±11.55^a^	66.09±11.05^a^	.847
**Education (yr)**	9.54±5.08^a^	10.45±5.24^a^	.606
**SGDS (maximum score = 15)**	1.18±1.25^a^	0.81±0.98^a^	.519
**K-MMSE (maximum score = 30)**	28.72±0.90^a^	28.72±1.10^a^	.949
**STAND (maximum score = 20)**	19.63±0.50^a^	19.27±0.90^a^	.478
Mean length of the conversation (second)	1225.91±670.73 ^a^	1260.27±417.86 ^a^	.748

^a^ Values are means ± SD; Mann-Whitney U test was conducted.

HA = Healthy adults; RHD = Right hemisphere damage.

SGDS = Short from Geriatric Depression Scale; K-MMSE = Korean version-Mini Mental State Exam; STAND = Screening Test for Aphasia & Neurologic-communication Disorders

**Fig 1:**
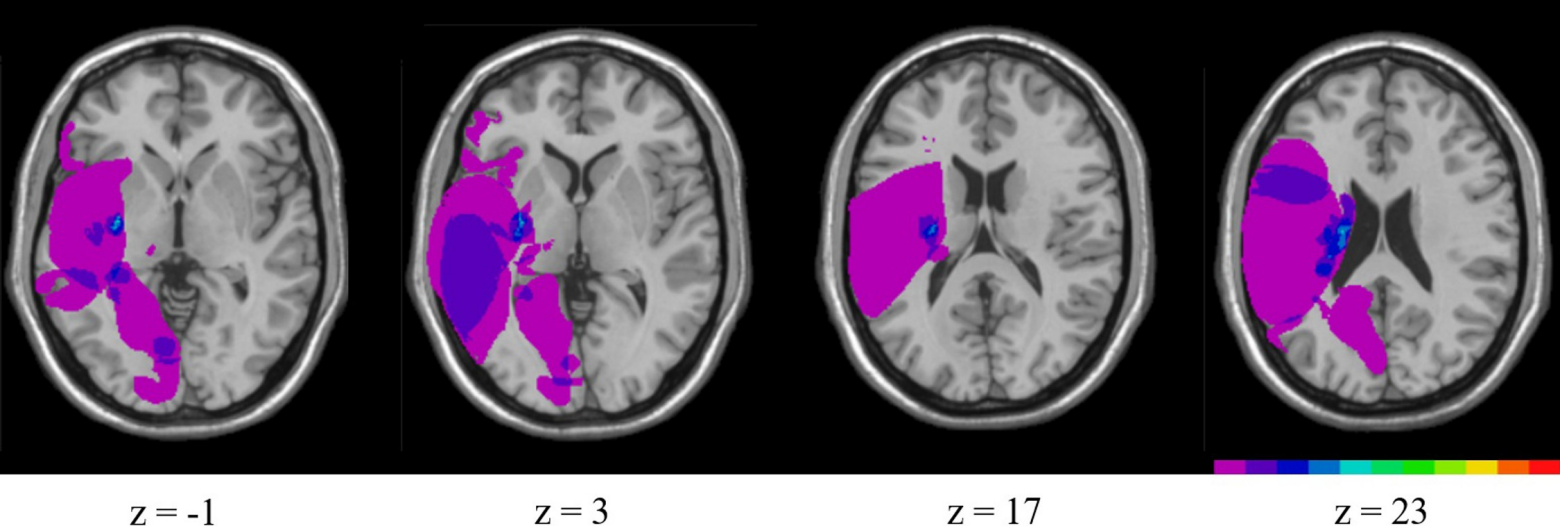
Lesion overlap maps for patients with right hemispheric damage (N = 11). The color bar indicates the number of participants with damage, while the numbers below indicate the corresponding MNI coordinates. (fuchsia = 1; red = 11). The areas of greatest lesion overlap extend in the vertical dimension from z = -19 to z = 47.

The lesion overlap maps for the RHD group generated with the MRIcro software are shown in Fig 1. The color scale indicates the number of patients with damage. Fuchsia color indicates one participant and red color indicates 11 participants overlapping. The most common sites of damage were in the subcortical area, especially the periventricular white matter. Regarding the cortical areas, the lesions were distributed among the inferior frontal lobe, lateral temporal lobe, lateral and medial parietal lobes, and medial occipital lobe. The affected lobes for each patient are presented in S1 Appendix. However, the site of the lesion was not consistent across most participants.

### Conversational participation indices between groups

There was no significant difference in the conversational participation index between the two groups (Table 3). The data distribution for the conversational participation index is presented in S2 Appendix. In addition, there was no difference in the conversational participation index between the subcortical and cortical groups within the RHD group (S2 Table in S1 File).

**Table 3:** Comparison of conversational participation indices.

	RHD	HA	*p*
**Number of turns**	46.00 (35.00,63.00)	61.00 (48.00,64.00)	.151
**Number of utterances per turn**	1.98 (1.35,3.71)	2.12 (1.79,2.21)	.699

Mann-Whitney U test was conducted. Values are median (interquartile range)

RHD = Right hemisphere damage; HA = Healthy adults

### Topic manipulation indices between groups

According to the topic manipulation index, the RHD group had a lower topic manipulation ability, a higher total number of topics, and a lower number of turns per topic than the HA group. The RHD group also showed higher rates of topic initiation and switching compared to the HA group. The topic maintenance rate was lower in the RHD group than in the HA group (Table 4). The data distribution for the topic manipulation index is presented in S2 Appendix. There was no difference in the topic manipulation index between the subcortical and the cortical groups within the RHD group (S2 Table in S1 File).

**Table 4:** Comparison of topic manipulation indices.

	RHD	HA	*P*
**Total number of topics**	18.00 (15.00,21.00)	13.00 (11.00,14.00)	.001
**Number of turns per topic**	2.57 (2.00,3.65)	4.61(4.07,5.42)	.003
**% of topic initiation**	4.65 (3.17,6.25)	3.17 (3.03,3.77)	.047
**% of topic maintenance**	86.04 (71.87,89.09)	92.85 (90.56,95.31)	.001
**% of topic switching**	9.30 (7.27,21.80)	1.66 (0,5.66)	< .0001

Mann-Whitney U test was conducted. Values are median (interquartile range)

RHD = Right hemisphere damage; HA = healthy adults

### Conversational breakdown indices between groups

The conversational breakdown index showed that the RHD group had a higher rate of overlap than the HA group. There was no significant difference in the rate of discontinuances between the two groups (Table 5), and no difference in the conversational breakdown index between the subcortical and the cortical group within the RHD group (S2 Table in S1 File). The data distribution for the conversational breakdown index is presented in S2 Appendix.

**Table 5:** Comparison of conversational breakdown indices.

	RHD	HA	*p*
**% of overlap**	1.85 (0, 6.66)	0 (0,0)	.040
**% of discontinuance**	0 (0,3.17)	0 (0,0)	.171

Mann-Whitney U test was conducted. Values are median (interquartile range)

RHD = Right hemisphere damage; HA = healthy adults

## Discussion

### Summary of the results

In this study, we compared the conversation characteristics in a group of patients with RHD with those in a healthy control group based on three indices—the conversational participation index, the topic manipulation index, and the conversational breakdown index. The comparison of topic manipulation index revealed that the RHD group had a lower topic maintenance rate than the HA group, but that topic initiation and topic switching rates were higher in patients with RHD. The conversational breakdown index also revealed that the RHD group showed higher rates of overlap than the HA group.

### Interpretation of the results

#### Conversation participation index

We found no significant difference between the RHD and HA groups with respect to the conversation participation index, which assessed the number of turns and the number of utterances per turn. Previous studies have shown inconsistent results. Patients with RHD showed a greater number of utterances than healthy participants in one study [8], while in other studies, patients showed a higher number of turns but fewer words per turn [46, 47]. The inconsistencies in these previous results might be due to differences in conversation topics. A conversation involves the expression of thoughts or feelings according to a specific context and topic. Therefore, the number of utterances can increase or decrease depending on the topic of the conversation [48]. The heterogeneity of the population might also be a factor in discrepancies in previous results. Studies examining conversational characteristics of patients with RHD reported heterogeneity among patients with RHD [46]. In particular, as these studies investigated the characteristics of small number (10–12) of RHD patients, it might have been difficult for these studies to show consistent results because the effects of population heterogeneity can be relatively pronounced in small-population studies [8, 47]. A visual inspection of the contents of the conversations in our study showed that some participants had more utterances across all subjects or more utterances on certain topic than others. Since this may be reflected in a reduced or normal level of conversation participation depending on the topic, previous studies also reported that it was difficult to assess the characteristics of patients with RHD by looking at their degree of conversation participation alone [8]. Furthermore, due to the task’s protocol, examiner participation was minimal and the participants’ conversations tended to be very one-sided. Thus, the conversational participation index may not be very sensitive.

#### Topic manipulation index

On assessment of the topic manipulation index, we found that RHD group participants frequently changed the topic, even within their turn, when they were supposed to talk about one specific topic. Normally, in order for the speaker to maintain a topic within the conversational turn, the context of the previous as well as the next utterance must be maintained. Frequent initiation of new topics or switching of topics could be perceived as being out of context from the listener’s point of view, making it difficult to understand the context of the conversation. Additionally, since patients with RHD often provide unnecessary details, insufficient content, or broadly related but not specifically appropriate information, their speech lacks relevance [14]. Patients with RHD are therefore described as being hyperfluent, but having topic manipulation difficulties [49]. Such patients might also have inferencing deficits [50], which makes it difficult for them to manipulate the discourse, as their assessment of their conversation partners’ needs is impaired [14]. Previous studies have also shown that RHD patients have difficulty maintaining a topic, judging appropriateness in a conversation, and considering the perspective of the listener [18, 51].

#### Conversational breakdown index

With respect to the conversational breakdown index, the RHD group had higher overlap rates than the HA group. In order to avoid overlap in a conversation, one needs to wait until the other’s turn has ended. In general, speakers may use specific utterances (syntactic or semantic) for turn endings while simultaneously using prosodic factors such as a decrease in speed or pitch [52, 53]. Therefore, to recognize that a partner’s turn has come to an end, the listener needs to be aware of not only syntactic and semantic signals, but also prosodic and pragmatic signals to catch the correct timing [54]. In prosody, the rhythm provides information on the interpretation of the language, and a proper understanding of intonation may help with clearer communication, as well as with grasping the context and associated emotions in a specific situation. Rhythm and intonation not only allude to the beginning and end of a conversation, but also make it easier to understand the order as well as turns in the conversation. Consequently, understanding of prosody is closely related to pragmatic language ability [55]. However, patients with RHD might have impaired prosodic processing ability [56] and difficulties using non-verbal information [46, 57]. For this reason, the frequent conversation overlaps observed in the RHD group may be due to impaired prosody and interpretation of indirect speech acts, which may interfere with turn end detection. Conversely, Riou reported that while prosody contributes to signaling for a topic shift, it is not established as critical for understanding topic ending [58]. Therefore, the results of this study must be interpreted with caution until they are validated through a clear confirmation of the relationship between aprosodia and topic-ending detection in RHD. Furthermore, the examiner deliberately produced very little and only relatively neutral speech. Due to such procedural characteristics, overlaps inevitably appeared infrequently; this may be why no overlap was noted in the HA group. In terms of diversity, overlaps are often behaviors with a positive affiliative impact that cannot be measured routinely or described as “inappropriate.” The RHD group’s non-zero tendency for overlaps needs to be interpreted carefully in this context.

#### Potential explanations of the conversational disorder in patients with RHD with acute cerebral infarction

In a previous study using fMRI, the right hemisphere was found to be more involved in conversational topic manipulation than the left hemisphere. During a topic maintenance task, the right hemisphere homologues of Broca’s and Wernicke’s area, dorsolateral prefrontal cortex, and cerebellum were activated [59]. Another fMRI study also showed that the right hemisphere was involved in information integration and discourse comprehension [60, 61], which we assumed was one of the reasons why patients with RHD showed impaired topic manipulation. Although both hemispheres are implicated in macrolinguistic processes during discourse, patients with RHD have been found to have greater impairment of coherence and construction of macropropositions [62]. fMRI studies have shown that the right temporal and frontal areas are activated when individuals perform tasks involving prosody. In particular, for the time-sensitive evaluation of prosody, the premotor cortex and inferior frontal gyrus in the right hemisphere are activated [63]. Impairments of prosody might also have affected the conversational task performance of patients with RHD in the current study.

We found no significant difference between patients who had only a subcortical lesion and those who had both cortical and subcortical lesions. Although most cortical and subcortical lesions did not overlap, the periventricular white matter area was found to overlap across patients. Periventricular white matter is white matter within 10 mm of the ventricular surface [64], including long white matter tracts. Therefore, we suggest that damage to the subcortex that connects cortical areas is as important as damage to a specific cortical area in the conversational disorder caused by RHD.

Patients with RHD show not only communication disorders, but also cognitive disorders [65], and the latter may also have influenced our findings. One study reported that patients with RHD showed attention deficits, which could affect discourse behavior compared to people without RHD [66]. In addition, since attention deficits are often accompanied by emotional deficits, it is difficult for such patients to interpret prosodic features [67]. Therefore, the higher rate of overlap without specific cortical lesion involvement, as revealed in this study, might be linked to the reduction in function of the prosody-related region due to a periventricular white matter lesion. Moreover, patients with RHD have also shown impairments of working memory [4]. In particular, right frontal function was found to be responsible for the processing of working memory [68], which is also closely related to communication problems in patients with an injured right hemisphere [69–71]. In order to properly maintain the topic of a conversation, it is necessary to understand the topic at hand, remember the content and context of the entire utterance, and produce the utterance appropriate to that context. This simultaneous information processing is based on working memory, a system that temporarily stores and integrates contents during a discourse [72]. However, for topic manipulation, brain connectivity may be more important than the function of a specific brain region.

#### Lack of structured tests to diagnose conversational disorders in patients with RHD

Our patients with RHD complained of difficulties in pragmatics and conversational discourse, but they had not previously been screened for language function, which is usually done by assessing left hemisphere-oriented language function. There exists a battery of tests to evaluate the cognitive-communication abilities of patients with RHD, such as the Mini-Inventory of Right Brain Injury-2 [73], the Right Hemisphere Language Battery [74], the Right Hemisphere Communication Battery [75], and the Protocole Montreal d’Evaluation de la Communication [76]. However, these tests mainly focus on figurative language, humor, and intonation; there are only few tests that include entire discourse or conversational aspects. In this study, we have thus investigated aspects of impaired conversation abilities in patients with RHD using a structured conversation task. Our findings might provide new clinical insight into the effect of RHD on conversation abilities.

### Limitations

This study has several limitations. First, the numerical values of the study results showed a greater standard deviation for the RHD group than for the HA group. While the type of cerebrovascular disorder and number of days after the onset of the disease were controlled for, the lesion size and site of lesion were not. As such, any such discrepancies may have affected the standard deviation of the patient group [77]. Second, as the large study of Ferré et al. [78] demonstrated, there is a subset of people with RHD who do not present with obvious communicative deficits. We therefore reviewed all individual data from the RHD group in our study, and found that none of the 11 patients performed as well as the HA group. However, since the number of patients with RHD was small, a clear process for generalizing and establishing conversational characteristics of RHD through a large-scale study is warranted in the future. Third, we did not evaluate sub-cognitive functions in detail by performing a comprehensive cognitive function test on our patients. It is thus possible that, unbeknownst to us, other cognitive functions (e.g., attention, working memory, and neglect), and not language functions, might have influenced conversational characteristics in this study. Further research including detailed neuropsychological test results is needed to examine the relationship between right hemisphere-related cognitive function and conversation characteristics. Fourth, this study showed behavioral differences and identified lesion locations in the RHD patient group. Future studies including patients with brain lesions but no symptoms are warranted, for a more complex analysis, in order to explain the underlying mechanisms of the conversational problems investigated here. Fifth, other researchers might have different ideas about how the term “structured conversation task” should be used. Thus, research-based formal and consensus-driven definitions should be considered in future studies.

### Clinical implications

This study investigated conversational disorder in patients with RHD in the acute stage of stroke. In these patients, topic manipulation impairments and conversation breakdowns occurred. These deficiencies may be one reason why communication is difficult between them and their interlocutors in a variety of communication situations. These impairments also occurred with or without cortical involvement. Therefore, our study suggests that we should reconsider the common misconception that the language function of patients with RHD is unaffected, even if their basic language assessment is in the normal range.

## Supporting information

S1 Appendix Information of RHD patients. (DOCX)

S2 Appendix Data distribution of HA and RHD patients. (DOCX)

S1 File (DOCX)
