# Supplementary material for: Effect of right hemispheric damage on structured spoken conversation
Source: PLoS One. 2022 Aug 11;17(8):e0271727. doi: 10.1371/journal.pone.0271727 (PMC9371334; doi:10.1371/journal.pone.0271727)
Supplement: S1 Appendix — (DOCX) [file pone.0271727.s001.docx]

**Appendix** 1. Information of RHD patients

| Gender | Age(yr) | Edu(yr) | MMSE | GDS | STAND | POT(day) | Lesion |
| --- | --- | --- | --- | --- | --- | --- | --- |
| M | 55 | 18 | 30 | 2 | 20 | 1 | Rt. BG infarction  Subcortex |
| M | 55 | 12 | 28 | 2 | 20 | 4 | Rt. MCA infarction  Frontal lobe |
| M | 61 | 11 | 29 | 0 | 20 | 4 | Rt. thalamic infarction  Subcortex |
| M | 63 | 12 | 28 | 1 | 19 | 7 | Rt. MCA infarction  Temporal lobe |
| F | 57 | 16 | 29 | 3 | 20 | 2 | Rt. cerebral infarction  Subcortex |
| F | 60 | 12 | 30 | 0 | 19 | 0 | Rt. PCA infarction  Temporal, Occipital lobe |
| F | 64 | 6 | 29 | 0 | 20 | 3 | Rt. BG, CR infarction  Subcortex |
| F | 77 | 4 | 27 | 2 | 20 | 3 | Rt. BG infarction  Subcortex |
| F | 82 | 2 | 29 | 0 | 19 | 6 | Rt. MCA infarction  Frontal, Temporal, Parietal lobe |
| F | 83 | 6 | 29 | 0 | 20 | 5 | Rt. CR infarction  Subcortex |
| F | 83 | 6 | 28 | 3 | 19 | 7 | Rt. PCA infarction  Temporal, Parietal, Occipital lobe |

Edu=education level; MMSE=Mini Mental State Examination; GDS=Geriatric Depression Scale; STAND= Screening Test for Aphasia and Neurologic-communication Disorders; POT=Post onset time(day); M=male; F=female; Rt=Right; BG=Basal Ganglia; MCA=Middle Cerebral Artery; PCA=Posterior Cerebral Artery; CR=Corona Radiata
