# Supplementary figures and images for: Effect of right hemispheric damage on structured spoken conversation
Source: PLoS One. 2022 Aug 11;17(8):e0271727. doi: 10.1371/journal.pone.0271727 (PMC9371334; doi:10.1371/journal.pone.0271727)

**Appendix** 2. Data distribution of HA and RHD patients


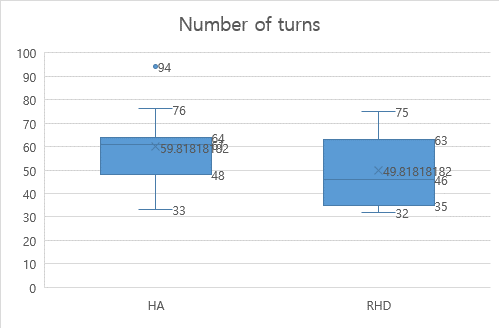


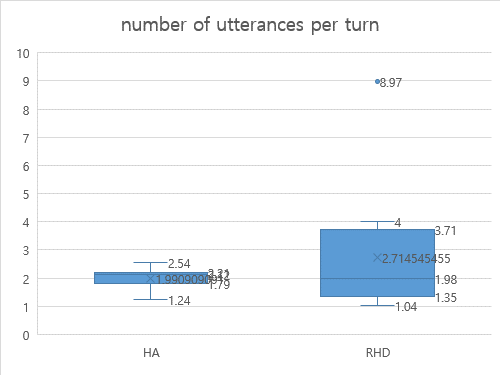


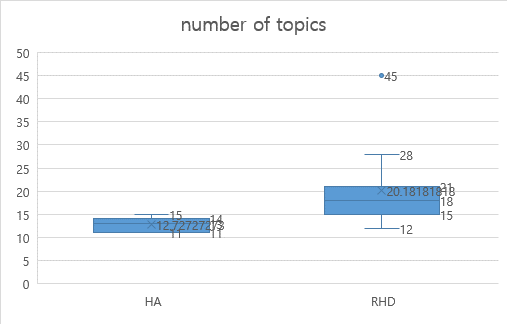


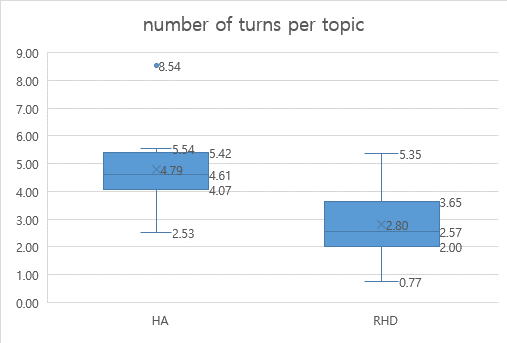


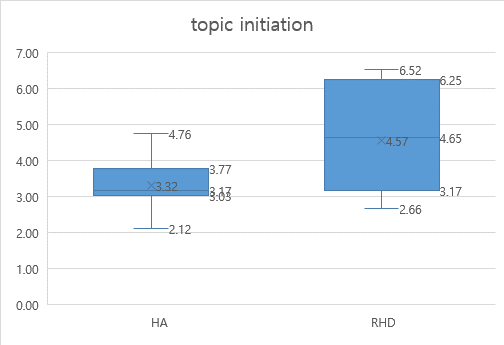


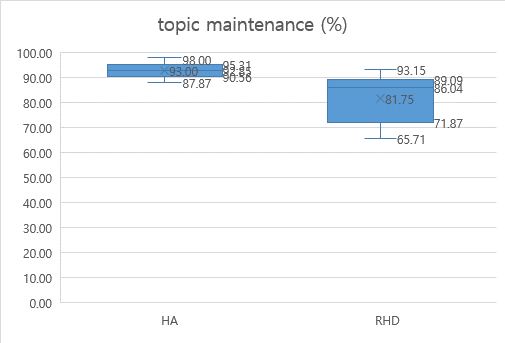


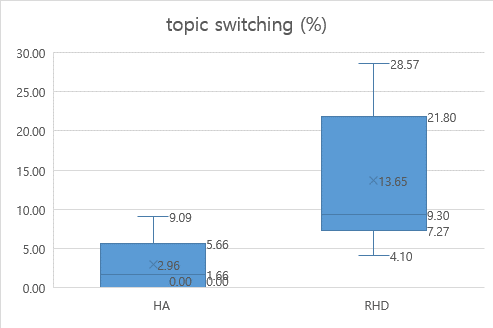


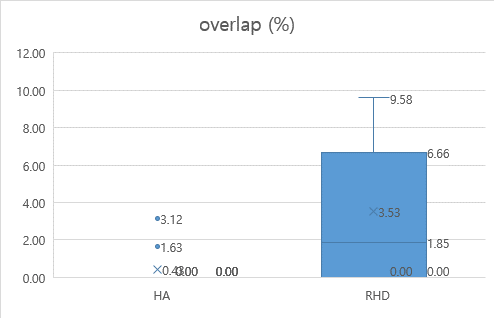


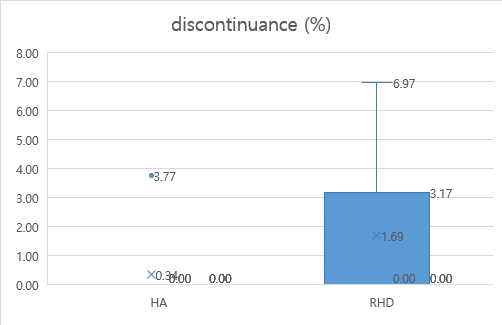

Supplement: S2 Appendix — (DOCX) [file pone.0271727.s002.docx]
