# Supplementary material for: Effect of right hemispheric damage on structured spoken conversation
Source: PLoS One. 2022 Aug 11;17(8):e0271727. doi: 10.1371/journal.pone.0271727 (PMC9371334; doi:10.1371/journal.pone.0271727)
Supplement: S1 File — (DOCX) [file pone.0271727.s003.docx]

**S1 Table. Criteria for utterance, turn, and topic.**

| Definition of utterance (Kim [1], Owens [2]) | |
| --- | --- |
|  | 1. One sentence is assumed to be one utterance. 2. A utterance is considered finished when time elapses (more than about 5 seconds), when there is a change of the topic, or when there is a marked change in prosody. 3. Even if the same word is expressed in a different context or situation, or if it is expressed in a new meaning, it is treated as a different utterance. 4. When the participant voluntarily corrects the utterance or repeats a word or phrase without adding new meaning, it is assumed to be one utterance (the part is marked with parentheses and excluded from the analysis). 5. Fillers used by the participant habitually are excluded from the analysis. 6. A meaningless sound or exclamation used to continue a sentence is excluded from the analysis. 7. Automated speech (e.g., counting, singing) is not considered an utterance and is excluded from analysis. However, if automatic speech is embedded in the utterance, it is included in the analysis. 8. In sentences that continue to connect using 'and' or a connecting ending '-ko', only one ‘and’ or only one ‘-ko’ should be included in one utterance. |
| Definition of turn (Heo & Lee [3], Kim & Lee [4], Adams & Bishop [5], Bishop, Chan, Adams, Hartley, & Weir [6], Bryan, Donahue, Pearl, & Sturm [7], Schober-Peterson & Johnson [8], Lee & Kim [9], Crystal, Fletcher, & Garman [10], Weiner & Goodenough [11] | |
|  | 1. Turn refers to all utterances continued by the same speaker, from when the speaker starts speaking until the conversation partner starts speaking. 2. If the topic of the conversation is changed while the participant is speaking, one turn is considered finished. 3. When continuing one’s turn on the same topic, if a pause of 3 seconds or more appears during an utterance, it is considered as a different turn. 4. Even if the participant speaks with words or phrases such as back-channel responses in his/her turn, it is considered as one turn. 5. As an indication that the conversation partner is listening, when the participant performs non-verbal actions such as shaking his/her head, nodding his/her head, and pointing, it is not regarded as a turn. However, in the case of such a verbal action in response to the conversation partner's question or instruction, it is regarded as a turn. |
| Definition of topic (Park, Choi, & Lee [12], Schober-Peterson & Johnson [8]) | |
|  | 1. A topic is the main information provided by the speaker through utterance, and it means a single utterance or a series of utterances related to each other with the same vocabulary, theme, concept, and components. 2. After a new topic is started, utterances that continue before being changed to a new topic are considered as one topic. 3. If content is added or elaborated on an already mentioned topic, it is not counted as a separate topic. 4. Even if one topic is terminated, changed to another topic, and the previous topic is mentioned again, it is not counted as a new topic. |

**S2 Table.** Comparison between the cortical and subcortical groups

|  |  | Cortical group (N=5) | Subcortical group (N=6) | *p* |
| --- | --- | --- | --- | --- |
| Demographic information | |  |  |  |
|  | Age | 63.00 (57.50,82.50) | 62.5 (56.5,78.5) | .931 |
|  | Education | 12.00 (4.00,12.00) | 8.50 (5.50,16.5) | .931 |
|  | SGDS | 1.00 (0,2.50) | 1.00 (0,2.25) | .931 |
|  | K-MMSE | 28.00 (28.00,29.50) | 29.00 (28.50,29.25) | .537 |
|  | STAND | 19.00 (19.00,19.50) | 20.00 (20.00,20.00) | .030 |
| Indexes of conversation | |  |  |  |
|  | Number of turns | 55.00 (39.00,74.00) | 41.50 (34.25,56.25) | .247 |
|  | Number of utterances per turn | 19.98 (1.37,2.93) | 1.96 (1.27,5.24) | .931 |
|  | Total number of topics | 18.00 (14.50,19.00) | 18.50 (14.25,32.25) | .537 |
|  | Number of turns per topic | 3.65 (2.16,4.51) | 2.41 (1.69,2.89) | .247 |
|  | % of topic initiation | 3.63 (2.70,6.39) | 4.83 (3.57,5.85) | .662 |
|  | % of topic maintenance | 89.09 (72.86,91.91) | 84.29 (70.33,87.15) | .329 |
|  | % of topic switching | 7.27 (5.38,20.68) | 11.79 (8.81, 23.55) | .247 |
|  | % of overlap | 6.66 (2.73,9.14) | .79 (0,2.64) | .052 |
|  | % of discontinuance | 0 (0,1.75) | 1.59 (0,5.49) | .429 |

Mann-Whitney U test was conducted. Values are median (interquartile range)

SGDS=Short from Geriatric Depression Scale; K-MMSE=Korean version-Mini Mental State Exam;

STAND=Screening Test for Aphasia & Neurologic-communication Disorders

**References for supporting information**

1. Kim, Y. T. (1997). Study of MLU in Korean 2-4 years children. *Korean Jour­nal of Communication & Disorders, 2,* 5-25.

2. Owens, R. E. (2004). *Language disorder: a functional approach to assessment and intervention* (4th ed.). Boston, MA: Allyn & Bacon.

3. Heo, H., & Lee, Y. (2012). Conversational turn-taking and topic manipula­tion skills in conversations of school-age low-achievers in language learn­ing. *Korean Journal of Communication & Disorders, 17,* 66-78.

4. Kim, S. E., & Lee, Y. (2007). Conversation characteristics of children with at­tention deficit hyperactivity disorder. *Korean Journal of Communication & Disorders, 12,* 662-675.

5. Adams, C., & Bishop, D. V. (1989). Conversational characteristics of children with semantic-pragmatic disorder. I. Exchange structure, turntaking, re­pairs and cohesion. *British Journal of Disorders of Communication, 24,* 211-239.

6. Bishop, D. V., Chan, J., Adams, C., Hartley, J., & Weir, F. (2000). Conversation­al responsiveness in specific language impairment: evidence of dispropor­tionate pragmatic difficulties in a subset of children. *Development and Psy­chopathology, 12,* 177-199.

7. Bryan, T., Donahue, M., Pearl, R., & Sturm, C. (1981). Learning disabled chil­dren’s conversational skills: the ‘TV Talk Show’. *Learning Disability Quar­terly, 4,* 250-259.

8. Schober-Peterson, D., & Johnson, C. J. (1993). The performance of eight-to ten-year-olds on measures of conversational skilfulness. *First Language, 13,* 249-269.

9. Lee, H. J., & Kim, Y. T. (2001). Turn-taking characteristics of children with specific language impairment and normal children. *Korean Journal of Com­munication & Disorders, 6,* 293-312.

10. Crystal, D., Fletcher, P., & Garman, M. (1976). *The grammatical analysis of language disability: a procedure for assessment and remediation.* London: Edward Arnold.

11. Weiner, S. L., & Goodenough, D. R. (1977). A move toward a psychology of conversation. *Discourse Production and Comprehension, 1,* 213-224.

12. Park, Y. J., Choi, J. E., & Lee, Y. (2017). Development of topic management skills in conversation of school-aged children. *Communication Sciences & Disorders, 22,* 25-34.
